# Deep Insight Into Long Non-coding RNA and mRNA Transcriptome Profiling in HepG2 Cells Expressing Genotype IV Swine Hepatitis E Virus ORF3

**DOI:** 10.3389/fvets.2021.625609

**Published:** 2021-04-29

**Authors:** Hanwei Jiao, Xuehong Shuai, Yichen Luo, Zhixiong Zhou, Yu Zhao, Bowen Li, Guojing Gu, Wenjie Li, Mengjuan Li, Hui Zeng, Xiaoyi Guo, Yu Xiao, Zhenhui Song, Ling Gan, Qingzhou Huang

**Affiliations:** ^1^College of Veterinary Medicine, Southwest University, Chongqing, China; ^2^Immunology Research Center, Medical Research Institute, Southwest University, Chongqing, China; ^3^Chongqing Veterinary Scientific Engineering Research Center, Southwest University, Chongqing, China; ^4^Institute of Animal Husbandry and Veterinary Medicine of Guizhou Academy of Agricultural Science, Guiyang, China

**Keywords:** swine HE, swine HEV, lncRNA, HepG2, ORF3

## Abstract

Swine hepatitis E (swine HE) is a new type of zoonotic infectious disease caused by the swine hepatitis E virus (swine HEV). Open reading frame 3 (ORF3) is an important virulent protein of swine HEV, but its function still is mainly unclear. In this study, we generated adenoviruses ADV4-ORF3 and ADV4 negative control (ADV4-NC), which successfully mediated overexpression of enhanced green fluorescent protein (EGFP)-ORF3 and EGFP, respectively, in HepG2 cells. High-throughput sequencing was used to screen for differentially expressed long non-coding RNAs (lncRNAs) and messenger RNAs (mRNAs). The *cis*-target genes of lncRNAs were predicted, functional enrichment (Gene Ontology [GO] and Kyoto Encyclopedia of Genes and Genomes [KEGG]) was performed, and 12 lncRNAs with statistically significant different expressions (*p* ≤ 0.05 and *q* ≤ 1) were selected for further quantitative real-time reverse transcription (qRT-PCR) validation. In HepG2 cells, we identified 62 significantly differentially expressed genes (DEGs) (6,564 transcripts) and 319 lncRNAs (124 known lncRNAs and 195 novel lncRNAs) that were affected by ORF3, which were involved in systemic lupus erythematosus, *Staphylococcus aureus* infection, signaling pathways pluripotency regulation of stem cells, the peroxisome proliferator-activated receptor (PPAR) signaling pathway, and platinum drug resistance pathways. *Cis*-target gene prediction identified 45 lncRNAs corresponding to candidate mRNAs, among which eight were validated by qRT-PCR: *LINC02476* (two transcripts), *RAP2C-AS1, AC016526, AL139099*, and *ZNF337-AS1* (3 transcripts). Our results revealed that the lncRNA profile in host cells affected by ORF3, swine HEV ORF3, might affect the pentose and glucuronate interconversions and mediate the formation of obstructive jaundice by influencing bile secretion, which will help to determine the function of ORF3 and the infection mechanism and treatment of swine HE.

## Introduction

Hepatitis E (HE) caused by the HE virus (HEV) is a new type of zoonotic infectious disease. HEV is mainly transmitted through the fecal–oral route, can cause acute hepatitis in humans, and is prevalent worldwide (1–3). About 20 million people are infected with HEV every year (4). In addition to the clinical symptoms of hepatitis, it also causes nerve, blood, and kidney diseases; neuralgia muscular atrophy; encephalitis; myelitis; and other diseases (5). Studies have shown that the continuous glycine motif in the β chain of the HEV-specific T-cell receptor (TCR) pathway might be the target for TCR binding heterozygosity recognition, thus promoting cross recognition, which might develop into a candidate for T-cell therapy for chronic HE (6).

Swine HE is caused by the swine HEV. In recent years, outbreaks of swine HE have appeared globally and have become a public health issue in various countries (7, 8). Swine HEV belongs to the family Hepeviridae and genus of HEV in the family of HEV (9). There were eight genotypes of HEV, and the main type of HEV in mainland China was type 1, followed by type 4. The host and route of transmission of different genotypes are completely different, and the symptoms after infection are also different. Its genome comprises about 7.6 kb, with a poly A tail structure at the 3′ end and three overlapping open reading frames (ORFs): ORF1, ORF2, and ORF3 (10, 11).

The ORF3 protein (hereafter referred to as ORF3) is a small and poorly characterized protein that is involved in the secretion of virus particles and other functions (12). Palmitoylation determines the subcellular location, membrane topology, and function of ORF3 in the life cycle of HEV (12). ORF3 forms polymer complexes related to endoplasmic reticulum (ER)-derived membranes through affinity interaction, which might be an attractive target for the development of antiviral drugs (13). ORF3 significantly inhibits the activity of the nuclear factor kappa B (NF-κB) signaling pathway, mediated by pattern-recognition receptors (PRRs), thus providing a breakthrough point for clarifying the function of ORF3 in chronic HEV infection and cirrhosis (14). ORF3 mediates the regulation of endosomal sorting complexes required for transport (ESCRT) through the “PSAP” motif (viral late domain) (15).

Long non-coding RNAs (lncRNAs) are transcripts longer than 200 nucleotides that lack functional ORFs, play an important role in many life activities, and have become a hot spot in genetics research (16, 17). The relationship between ORF3 and long non-coding RNAs (lncRNAs) has not been reported. Although the function of HEV ORF3 has been studied, the function of swine HEV ORF3 remains unclear. In the present study, we used adenovirus-mediated overexpression of genotype IV swine HEV ORF3 in HepG2 cells derived from a 15-year-old white liver cancer tissue; the adherent HepG2 cells could be subcultured stably by 0.25% trypsin containing 0.01% EDTA, and transcriptome sequencing was performed to identify significantly differentially expressed lncRNAs and their target genes affected by ORF3, which form a basis to reveal the function of ORF3, explain the interaction mechanism between swine HEV and target cells, and provide a scientific basis for the prevention and treatment of swine HE.

## Materials and Methods

### Cell Lines

HepG2, 293A, and 293T cells were purchased from the Shanghai Cell Bank of the Chinese Academy of Science and cultured at 37°C, 5% CO_2_ in Dulbecco's modified Eagle's medium (DMEM) (Life Technology, Carlsbad, CA, USA) containing 10% fetal bovine serum (Life Technology) supplemented with 10% penicillin (100 U/ml) and streptomycin (100 μg/ml) (both Life Technology).

### Preparation of Recombinant Adenovirus ADV4-ORF3 and ADV4-NC

Sangon Biotech (Shanghai, China) Co., Ltd., synthesized the ORF3 gene (345 bp) of genotype 4 swine HEV. Adenoviruses ADV4-ORF3 and ADV4 negative control (ADV4-NC) were generated by the Shanghai GenePharma Co, Ltd (Shanghai, China). The enhanced green fluorescent protein (EGFP) was fused to the N-terminus of ORF3. Both of them were sequenced using Sanger sequencing, and no mutations compared with the published sequence of ORF3 and the rest of the plasmid were found. Briefly, *Eco*RI and *Bam*HI were added to the upstream and downstream primers of the target gene, respectively. Swine HEV ORF3 was cloned into vector ADV4 (cytomegalovirus [CMV]/IRES-green fluorescent protein [GFP]) (Supplementary Figure 1); this vector, with a molecular weight of 7,500 bp, comprises a CMV promoter, a multiple cloning site, GFP, IRES, an ampicillin-resistant gene, and the Ad5 skeleton. Highly pure and non-toxic recombinant expression shuttle plasmid ADV4-ORF3 and framework plasmid pGP-Ad-Pac were prepared. The shuttle plasmids ADV4-ORF3 and ADV4-NC and framework plasmid pGP-Ad-Pac (molecular weight of 35,000 bp, comprising an Ori promoter, pA; an ampicillin-resistant gene, dE3; and Ad5 skeleton) (Supplementary Figure 2) were co-transfected into 293A cells with the RNAi mate transfection reagent (GenePharma, Shanghai, China). Six hours after transfection, the culture medium was replaced with a complete medium, and the fresh medium was supplemented every 7 days. Then, the cells and supernatant were collected and placed in a centrifuge tube, frozen and thawed three times, and centrifuged at 800 × *g* for 5 min, and the supernatant was regarded as the primary adenovirus solution. After three successive generations of repeated amplification and collection of adenovirus, a large amount of adenovirus was obtained, which was then purified and concentrated to obtain a high titer of recombinant adenovirus concentrate. The method of virus purification and concentration is briefly described as follows: the adenovirus was purified through the CsCl density gradient centrifugation dialysis method, 2.0 ml of CsCl solution was added with a density of 1.40 g/ml, and then 3.0 ml of CsCl solution was slowly added with a density of 1.30 g/ml. Then 5 ml of the virus suspension was added, and then the dialysis solution was stirred at 4°C overnight.

The ADV4-ORF3 and ADV4-NC adenovirus titers were determined in 293T cells. Briefly, 293T cells were added to a 96-well plate at 10^4^ cells per well. The amount of medium added was 200 μl. The plate was incubated for 24 h, and the adenovirus to be tested was diluted to 10^−2^, 10^−3^, 10^−4^, 10^−5^, 10^−6^, and 10^−7^ with complete medium. After aspirating off the supernatant, the adenovirus solutions at different dilutions were added at 200 μl per well. Samples were repopulated, and the culture medium without adenovirus was set as a control. The plates were incubated at 37°C, 5% CO_2_. The culture continued for 36–48 h under CO_2_ conditions. The cytopathic effect (CPE) was observed using microscopy, and the virus titer was calculated according to a formula supplied by Baxter Healthcare Corporation (Round Lake, IL, USA).

### Infection Assay of Recombinant Adenovirus ADV4-ORF3 and ADV4-NC

HepG2 cells were seeded into a 12-well plate (1 × 10^6^ cells per well), infected with ADV4-ORF3 and ADV4-NC at 12 h post seeding at a multiplicity of infection (MOI) of 5:1. The cells were harvested after infection for 24 h. Then, 0.8 μl of polybrene was added to each well to improve the transfection efficiency. Triplicate infection experiments were performed. Quantitative reverse transcription PCR (qRT-PCR) was used to confirm ORF3 overexpression. The sequences of the primers were as follows:

ORF3: forward primer 5′-GCTCCTCCTGCTTTTGCCTA-3′ and reverse primer 5′-GCTGAGAATCAACCCGGTCA-3′ and
GAPDH: forward primer 5′-GCTCTCTGCTCCTCCTGTTC-3′ and reverse primer 5′-CCAAATCCGTTGACTCCGAC-3′.

The sample of ADV4-ORF3-infected cells was named Ad_ORF3, and that of ADV4-NC-infected cells was named Ad_GFP.

### Preparation of Polyclonal Antibodies Against ORF3 and Western Blot

Genotype IV swine HEV ORF3 gene was synthesized by Biotechnology (Shanghai) Co., Ltd; the relevant information of the virus strain used as a template for the ORF3 was referenced from Liu et al. (18). We designed the prokaryotic expression prime (forward primer: 5′-CGCTGAATTCATGGCGATGCCACCATGCG-3′; reverse primer: 5′-GCCTAAGCTTTCAGCGGCGAAGCCCCAG-3′) and used pET-28a(+) for the construction prokaryotic vector of pET28-ORF3. The recombinant plasmid was transformed into BL21 competent cells, which were induced by 0.1 mmol/L isopropyl β-d-1-thiogalactopyranoside (IPTG) for 6 h at 37°C, and the cell was broken by ultrasound to collect the ORF3 antigen. After SDS-PAGE, the target protein was cut to a target band of 12 kDa and used to immunize male New Zealand white rabbits. For the first immunization, 375 μg of ORF3 protein was used to inject rabbits; the same method was used for the second and third injections after another 14 days. Ten days after the last immunization, blood was collected from the heart to isolate the antiserum, and the titer of antiserum was detected by indirect enzyme-linked immunosorbent assay (ELISA).

HepG2 cells were infected with the high-titer recombinant adenovirus of ADV4-NC (control) and ADV4-ORF3 for 24 h. The cells were collected and lysed with radioimmunoprecipitation assay (RIPA) containing 1 μg/ml phenylmethylsulfonyl fluoride (PMSF). Forty micrograms of total protein was analyzed by 15% SDS-PAGE and electrically transferred to a polyvinylidene difluoride (PVDF) membrane for 1.5 h, which was blocked with 5% skimmed milk for 2 h, washed three times with TBST, and incubated with anti-ORF3 primary antibody (1:200 dilution) at 4°C overnight and a monoclonal mouse anti-β-actin (1:2,000 dilution; Santa Cruz Biotechnology, USA); the PVDF membrane was washed three times with TBST, incubated with horseradish peroxidase (HRP)-labeled goat anti-rabbit IgG (1:5,000 dilution; Abcam, USA) and mouse-IgGk BP-HRP (1:5,000 dilution; Santa Cruz Biotechnology, USA) for 2 h, and washed threes with TBST. The PVDF membrane was developed with SuperSignal™ West Pico PLUS Chemiluminescent Substrate kit (Thermo Scientific, USA), and the ORF3 and β-actin proteins were detected by CCD cameras.

### Indirect ELISA

The purified recombinant ORF3 protein was coated on the plate, coated at 4°C for 10 h, and washed four times with PBST. PBST containing 5% skimmed milk powder was added, 200 μl per well; sealed at 37°C for 2 h; and washed four times. The antiserum with dilution from 1:800 to 1:6,400 was serum; the serum before immunization was used as a negative control and washed four times with PBST at 37°C for 1 h. HRP-labeled protein A (HRP-SPA) was added as the second antibody and washed with PBST at 37°C for 1 h. Tetramethylbenzidine (TMB) was used to develop the color for 10 min, and the OD_450nm_ value was detected by an enzyme reader. A ratio of ≥2.1 for the OD_450nm_ value of the standard to be tested/the OD_450nm_ value of the negative control was regarded as positive, and the highest dilution of the positive reaction was regarded as the serum titer.

### RNA Sample Preparation and Transcriptome Sequencing

Total RNA was extracted and purified using the TRIzol reagent (Invitrogen, Carlsbad, CA, USA) following the manufacturer's procedure. The RNA amount and purity of Ad_GFP_1, Ad_GFP_2, Ad_GFP_3, Ad_ORF3_1, Ad_ORF3_2, and Ad_ORF3_3 samples were quantified using a NanoDrop ND-1000 instrument (NanoDrop, Wilmington, DE, USA). The RNA integrity was assessed using an Agilent 2100 with an RNA integrity number (RIN) >7.0. Approximately 5 μg of total RNA was used to deplete ribosomal RNA according to the protocol of the Ribo-Zero™ rRNA removal kit (Illumina, San Diego, CA, USA). After removal of the ribosomal RNAs, the remaining RNAs were fragmented into small pieces using divalent cations under high temperature. Then, the cleaved RNA fragments were reverse-transcribed to create the cDNA using ProtoScript II Reverse Transcriptase and First-Strand Synthesis Mix (Ambion, Austin, TX, USA), which were next used to synthesize U-labeled second-stranded DNAs with *Escherichia coli* DNA polymerase I, RNase H, and dUTP. An A-base was then added to the blunt ends of each strand, preparing them for ligation to the indexed adapters. Each adapter contained a T-base overhang for ligating to the A-tailed fragmented DNA. Single- or dual-index adapters were ligated to the fragments, and size selection was performed using AMPure XP beads. After heat-labile UDG enzyme treatment of the U-labeled second-stranded DNAs, the ligated products are amplified by PCR using the following conditions: initial denaturation at 95°C for 3 min, eight cycles of denaturation at 98°C for 15 s, annealing at 60°C for 15 s, an extension at 72°C for 30 s, and then a final extension at 72°C for 5 min. The average insert size for the final cDNA library was 300 bp (± 50 bp). Finally, we performed paired-end sequencing on an Illumina HiSeq 4000 sequencer (LC Bio, Hangzhou, China).

### Differentially Expressed lncRNA and mRNA Analyses

Firstly, Cutadapt (19) was used to remove the reads that contained adaptor contamination, low-quality bases, and undetermined bases. The sequence quality was then verified using FastQC (http://www.bioinformatics.babraham.ac.uk/projects/fastqc/). We used Bowtie 2 (20) and HISAT2 (21) to map reads to the human genome. The mapped reads of each sample were assembled using StringTie (22). Then, all the transcriptomes from Ad_GFP_1, Ad_GFP_2, Ad_GFP_3, Ad_ORF3_1, Ad_ORF3_2, and Ad_ORF3_3 samples were merged to reconstruct a comprehensive transcriptome using Perl scripts. After the final transcriptome was generated, StringTie and edgeR (23) were used to estimate the expression levels of all transcripts.

Transcripts that overlapped with known mRNAs and transcripts shorter than 200 bp were discarded. Then we utilized the coding potential calculator (CPC) (24) and coding–non-coding index (CNCI) (25) to predict transcripts with coding potential. All transcripts with a CPC score < −1 and a CNCI score <0 were removed. The remaining transcripts were considered as lncRNAs. Differential expression analysis of mRNAs and lncRNAs used StringTie to perform expression-level analyses for mRNAs and lncRNAs by calculating the fragments per kilobase of transcript per million mapped reads (FPKM) (26). FPKM can represent the expression of lncRNAs. In the present study, we identified five class codes defining lncRNAs: class code j indicated potentially novel isoforms (fragments), in which at least one splice junction is shared with a reference transcript; class code i indicated a transfragment falling entirely within a reference intron; class code o indicated a generic exonic overlap with a reference transcript; class code u indicated an unknown, intergenic transcript; and class code x indicated an exonic overlap with a reference on the opposite strand. The differentially expressed mRNAs and lncRNAs were selected using log2 (fold change) > 1 or log2 (fold change) < −1 and with statistical significance (*p* < 0.05) using the R package module edgeR (23).

### Target Gene Prediction and Functional Analysis of lncRNAs

To explore the function of the lncRNAs, we predicted their *cis*-target genes. In this study, coding genes in the 100 kb regions upstream and downstream of the lncRNAs were selected using a Python script. Then, we performed functional analysis of the lncRNA target genes using Blast2GO (27). Significance was expressed as a *p* < 0.05.

### qRT-PCR Validation

The primers were designed using the NCBI Primer online software. Approximately 1 μg of total RNA was used for reverse transcription using a PrimeScript™ RT reagent kit (Takara, Shiga, Japan), and validation by qRT-PCR was performed using TB Green® Premix Ex Taq™ II (Tli RNaseH Plus) (Takara). GAPDH was used as an internal control. The primers are listed in Table 1. The relative expression levels of lncRNAs and mRNAs were determined using the 2^−ΔΔCt^ method. Three independent replicates were performed. Student's *t*-test was used for statistical analysis. A *p* < 0.05 was considered statistically significant, indicated by an asterisk, and a *p* < 0.01 was considered highly statistically significant, indicated by two asterisks.

**Table 1:** Primers for the selected significantly differentially expressed lncRNAs (*p* ≤ 0.05 and *q* ≤ 1) for qRT-PCR validation.

**lncRNAs_name**	**Transcripts_name**	**Primer sequence (5'-3')**
PLCG1-AS19	ENST00000454626	F: CTGCCTCACAGGAGATCCAC
		R: CAGCCTTGGCCATCCTCATT
AL139099	ENST00000555043	F: CTTCCTTTGTCGCCCATTGC
		R: AGCTGTAGCTGCGCTCCC
AC005062	ENST00000449573	F: TACTGGAAGATGGCGGTTCC
		R:GAGACAGAAAGCGGAGTCTCA
FP236383	ENST00000625598	F: AACGCCACTTGTCCCTCTAA
		R: ACACGGACAGGATTGACAGA
FP671120	ENST00000627981	F: AACGCCACTTGTCCCTCTAA
		R: ACACGGACAGGATTGACAGA
FP671120	ENST00000631211	F: AACGCCACTTGTCCCTCTAA
		R: ACACGGACAGGATTGACAGA
ZNF337-AS1	ENST00000428254	F: AAGCGTGGTGTTCTTCCCTG
		R: CAGCATCCGCACAACAATGG
ZNF337-AS1	ENST00000455791	F: CCCGCGATCTGTCTCATTCC
		R: GGGCACAGGTAGGTGGTTAG
ZNF337-AS1	ENST00000420803	F: CAACTACCTCCCCTGTGCAA
		R: TTGGAGCCATCTTTCGAGGC
ZNF337-AS1	ENST00000421829	F: CTACCTCCCCTGTGCAAGTT
		R: GGCACAGGTAGGTGGTTAGC
ZNF337-AS1	ENST00000414393	F: CCCGCGATCTGTCTCATTCC
		R: TTGCACAGGGGAGGTAGTTG
ZNF337-AS1	ENST00000439498	F: TGGAATTCCCGCGATCTGTC
		R: GGGCACAGGTAGGTGGTTAG
AP005329	ENST00000581905	F: TCCTGTATGGTGCCTGGAGA
		R: AGGTGGACACCCTGTAGTTC
AP000977	ENST00000501964	F: GTGCATGCAGGTGGCATTAG
		R: GGGACAGAGGGGGATATGGT
LINC02476	ENST00000431071	F:ACTTTCCCTGGCAAACAAAAACA
		R:GCTTCCTAGGTAGGACAGGGA
LINC02476	ENST00000426413	F:GGTGTCGAGCTGTGAATGAGA
		R:ATGGGATGGGGCTGGGTTAT
RAP2C-AS1	ENST00000441399	F:ACTTAGCCGTGCCTGACAAA
		R:GCTCCAAAAAGGCACCCTTG
RAP2C-AS1	ENST00000421483	F:CAAGGGTGCCTTTTTGGAGC
		R:AAGAGCTTGATGACTCCGGC
AC107959	ENST00000502083	F:TGCTCTGTCTGGCCAAATCC
		R:CCCATGTGAAGCATTGCCTG
AC107959	ENST00000523884	F:TCTTCACCACCACCATCAGC
		R:GCCGAGCTTTTGTGAGCATC
AC016526	ENST00000554225	F:CACACTGGCCTTAGGGTGAC
		R:ACGTCTTTGTGAAGTCGGCA

## Results

### Recombinant Adenovirus-Mediated Overexpression of ORF3

**Figure 1:**
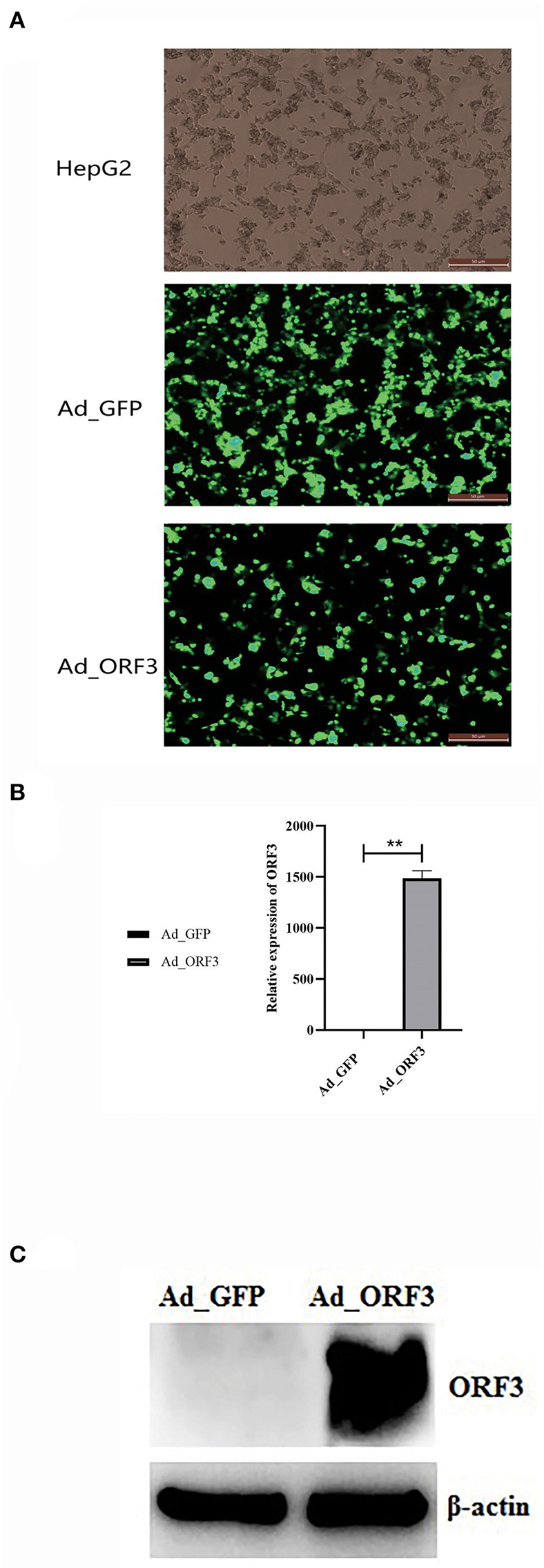
Adenovirus of ADV4-ORF3 and ADV4-NC mediated overexpression of ORF3 in HepG2 cells. **(A)** Fluorescence microscope observation of HepG2 cells infected by adenovirus of ADV4-ORF3 and ADV4-NC for 24 h. **(B)** qRT-PCR validation for the relative expression level of ORF3. **(C)** Western blot analysis of HepG2 cells infected by adenovirus of ADV4-ORF3 and ADV4-NC for 24 h with rabbit polyclonal antibody against ORF3. β-Actin was used as an internal control. *p < 0.05, **p < 0.01.

HepG2 cells were infected with the high-titer recombinant adenovirus of ADV4-NC (control) and ADV4-ORF3 for 24 h. EGFP expression was detected using fluorescence microscopy. The results showed that EGFP and the EGFP-ORF3 fusion protein were successfully expressed in HepG2 cells (Figure 1A). qRT-PCR was used to detect the relative expression of the ORF3 RNA, with *GAPDH* (encoding glyceraldehyde-3-phosphate dehydrogenase) used as the internal control. The results demonstrated that the recombinant adenovirus successfully mediated the overexpression of ORF3 (Figure 1B).

The indirect ELISA results showed that the polyclonal antibody titer reached 1:12,800. Western blot was performed on equal amounts of 40 μg protein extracted from Ad_GFP and Ad_ORF3. β-Actin was used as an internal control. With rabbit polyclonal antibodies against ORF3, the western blot results indicated that the specific band at a molecular weight of about 11.7 kDa for cell extracts from Ad_ORF3 was found, but no ORF3 expression in Ad_GFP was found (Figure 1C).

### DEGs and Transcript Cluster Analysis

We obtained raw data generated from the Illumina HiSeq 4000 sequencer. Cutadapt was used to filter out the unqualified sequence and get the clean reads (20) after the six samples (Ad_GFP_1, Ad_GFP_2, Ad_GFP_3, Ad_ORF3_1, Ad_ORF3_2, and Ad_ORF3_3) passed the quality control test. The mapped reads of each sample were assembled using Cufflinks (28). Then, all transcriptomes from the six samples were merged to reconstruct a comprehensive transcriptome using Cuffmerge. After the final transcriptome was generated, Cuffdiff was used to estimate the expression levels of all transcripts. In this study, the total mapped genes and transcripts were 58,825 and 208,460.

**Figure 2:**
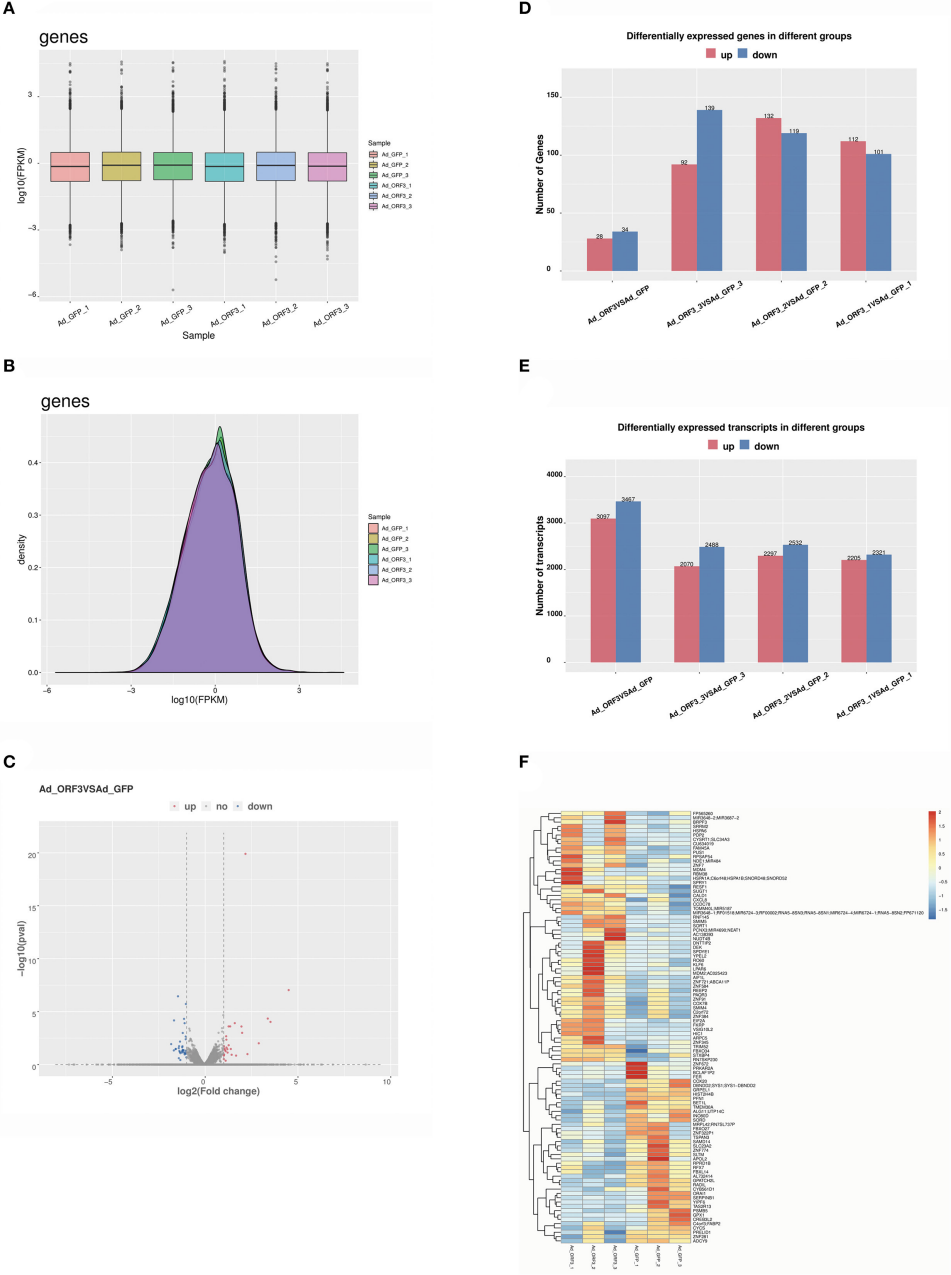
Analysis of DEGs and transcripts. **(A)** Distribution of gene expression values for each sample. The x-axis is the sample name, and the y-axis is log10 (FPKM). The box graph of each region corresponds to five statistics (the maximum, the upper quartile, the median, the lower quartile, and the minimum, respectively, from the top to the bottom). **(B)** Gene expression density distribution. The x-axis is the log10 (FPKM), and the y-axis shows the gene expression density. **(C)** Volcano analysis of differential expression levels of genes; the x-axis is log2 (fold change), which represents the variation of the differential expression of multiple genes in different samples, and the y-axis shows the –log10 (*p*-value), which represents the statistical significance of the change in the gene expression levels; red indicates upregulated DEGs, and dark blue indicates downregulated DEGs. **(D)** The statistics of the frequency of upregulation and downregulation of genes with significantly differential expressions. Red represents upregulated genes, and dark blue represents downregulated genes. **(E)** The statistics of the frequency of upregulated and downregulated transcripts with significant differential expression. Red represents upregulated transcripts, and dark blue represents downregulated transcripts. **(F)** Heatmap of partial DEGs; red indicates upregulated DEGs, and dark blue indicates downregulated DEGs.

The distribution statistics of genes in each sample was represented by FPKM box charts, and the expression levels of genes were interpreted from the analyses shown in Figure 2A. The gene expression density distribution of log10 (FPKM) could be used to compare the expression trend of different samples. In this study, the gene expression density distribution of each sample showed a normal distribution, and the gene expression trend of biological repeat samples were consistent (Figure 2B). All the differentially expressed genes (DEGs) were analyzed using volcano plots (29), which provided the overall distribution of the DEGs (Figure 2C). The results indicated that there were 62 significant DEGs, including 28 upregulated genes and 34 downregulated genes. We identified 6,564 significantly differentially expressed transcripts, including 3,097 upregulated transcripts and 3,467 downregulated transcripts (Figures 2D,E). Cluster analysis of the DEGs provided a visual display of the gene expression in each sample. To better reflect the cluster expression pattern, we used log10 (FPKM + 1) to display gene expression. At the same time, FPKM could be displayed by the *Z* value. The partial DEGs are shown in a heatmap, in which red indicates upregulated DEGs and dark blue indicates downregulated DEGs (Figure 2F).

### Analysis of Differentially Expressed lncRNAs

**Figure 3:**
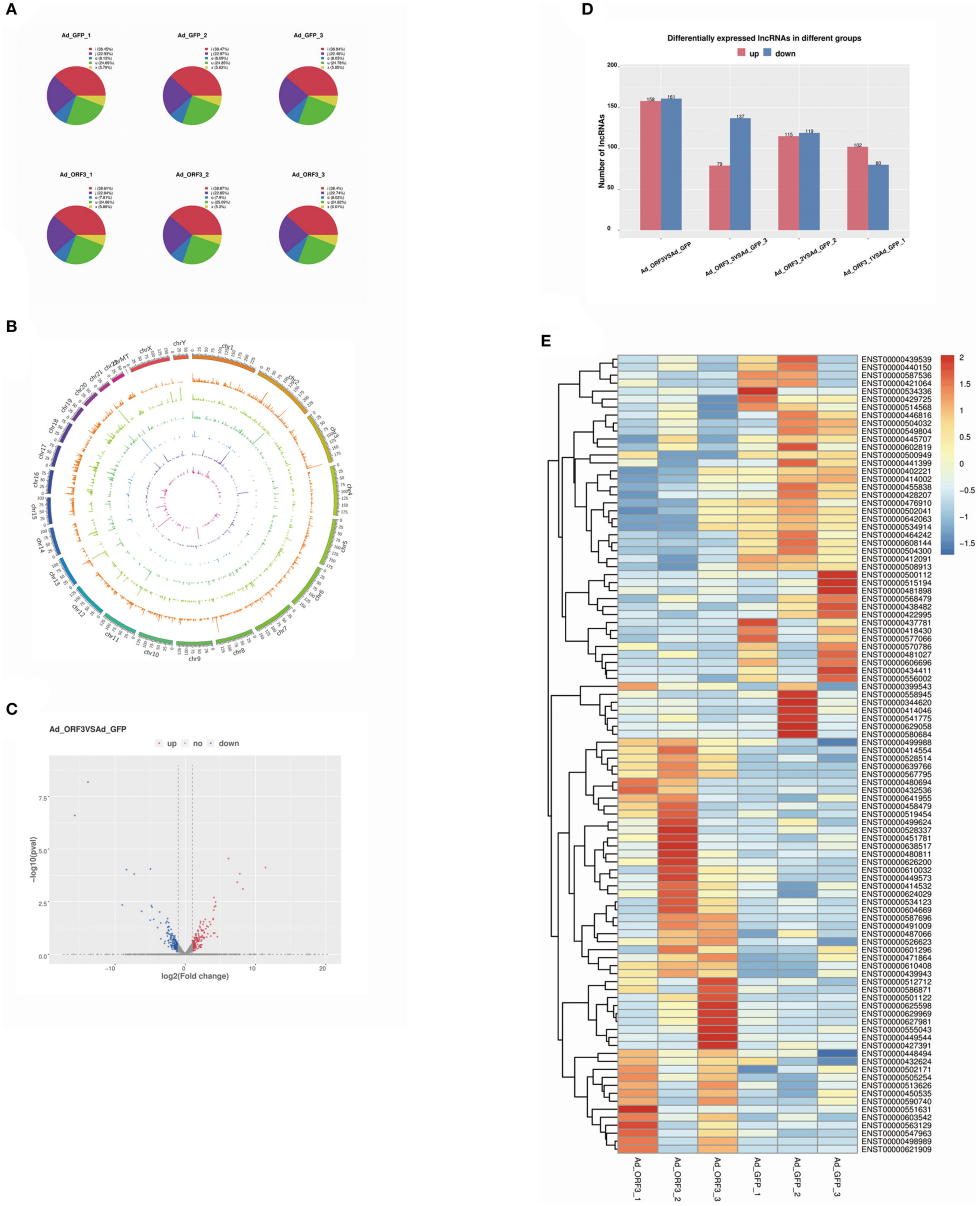
Analysis of differentially expressed lncRNAs. **(A)** Pie chart of different class code proportions of the lncRNAs in each sample. There are five class codes of lncRNA: j indicates a potentially novel isoform (fragment); i indicates a transfragment falling entirely within a reference intron; o indicates a generic exonic overlap with a reference transcript; u indicates an unknown, intergenic transcript; and x indicates an exonic overlap with the reference on the opposite strand. **(B)** Visualization results of lncRNAs from different samples, showing the distribution of lncRNA candidates in the chromosome more intuitively. **(C)** Volcano analysis of differentially expressed lncRNA levels; the x-axis is log2 (fold change), and the y-axis is –log10 (*p*-value). Red represents upregulated lncRNAs, and dark blue represents downregulated lncRNAs. **(D)** Statistics of the frequency of upregulated and downregulated lncRNAs with significant differential expression. Red represents upregulated lncRNAs, and dark blue represents downregulated lncRNAs. **(E)** Heatmap of differentially expressed lncRNAs; red represents upregulated lncRNAs, and dark blue represents downregulated lncRNAs.

The software StringTie was used to assemble the reads. Known mRNAs and transcripts <200 bp were removed, and then lncRNAs were predicted among the remaining transcripts. The prediction software used comprised CPC and CNCI. FPKM was used to measure the expression levels of the lncRNAs. There were five class codes of lncRNAs, namely, i, j, o, u, and x (Figure 3). The proportions of these five class codes of six samples (Ad_GFP_1, Ad_GFP_2, Ad_GFP_3, Ad_ORF3_1, Ad_ORF3_2, and Ad_ORF3_3) are shown in a pie chart (Figure 3A).

To show the distribution of lncRNA candidates in the chromosomes more intuitively, we used the software of Circos (www.circos.ca) to map the lncRNAs to the genome. This analysis was mainly divided into two parts. First, genome mapping was carried out according to the different classifications of the lncRNAs. Second, genome mapping was carried out according to the lncRNAs in the different samples. During mapping, each chromosome was analyzed every 25 mb as the basic unit. When the lncRNA-related genome in different samples was visualized as a map, the expression of the lncRNAs in each segment was counted. When the genome of different lncRNA types was visualized, the number of lncRNAs in each segment was counted (Figure 3B).

We identified 319 significantly differentially expressed lncRNAs, including 124 known lncRNAs and 195 novel lncRNAs. Among them, 158 lncRNAs were significantly upregulated and 161 lncRNAs were significantly downregulated (Figures 3C,D). In the follow-up study, we took the 124 known lncRNAs with significantly differential expressions as the research target, and the top 100 known lncRNAs were shown in a heatmap, in which red represented upregulated lncRNAs and dark blue represented downregulated lncRNAs (Figure 3E).

### Comparative Analysis of the Structural Characteristics of Differentially Expressed lncRNAs and mRNAs

**Figure 4:**
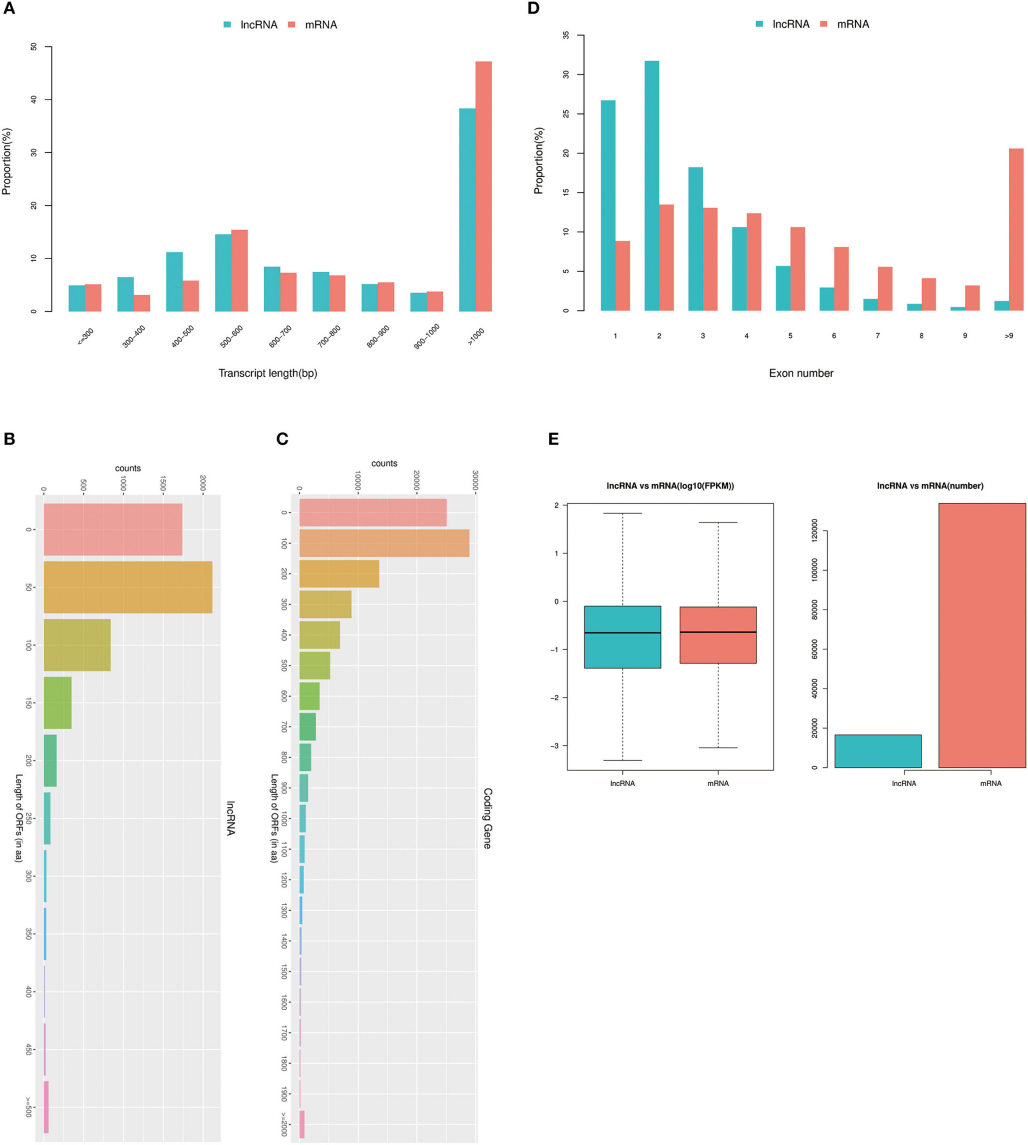
Analysis of the structural characteristics of differentially expressed lncRNAs and mRNAs. **(A)** lncRNA and mRNA length statistics and comparisons; the x-axis represents the transcript length, and the y-axis represents the proportion. **(B)** Distribution of the ORF lengths of lncRNAs. **(C)** Distribution of the ORF lengths of mRNAs. **(D)** Statistics of lncRNA and mRNA exons. The x-axis represents the exon number of the lncRNA and mRNA, and the y-axis represents the proportion. **(E)** lncRNA and mRNA expression levels were statistically analyzed from two levels of log10 (FPKM) and number.

It has been reported that the structural characteristics (length distribution, the number of exons, and ORF length) and expression levels of lncRNA and mRNA were very different (30). Therefore, this analysis mainly focused on the structural characteristics and expression levels of lncRNAs and mRNAs. lncRNA and mRNA length statistics and comparisons revealed the percentages of different lengths of transcripts (Figure 4A). The results show that there were 814 lncRNAs and 6,160 mRNAs (with transcript length of ≤300 bp), 1,076 lncRNAs and 3,722 mRNAs (with transcript length of 300–400 bp), 1,856 lncRNAs and 7,009 mRNAs (with transcript length of 400–500 bp), 2,415 lncRNAs and 18,600 mRNAs (with transcript length of 500–600 bp), 1,401 lncRNAs and 8,797 mRNAs (with transcript length of 600–700 bp), 1,239 lncRNAs and 8,225 mRNAs (with transcript length of 700–800 bp), 855 lncRNAs and 6,589 mRNAs (with transcript length of 800–900 bp), 581 lncRNAs and 4,555 mRNAs (with transcript length of 900–1,000 bp), and 6,356 lncRNAs and 56,900 mRNAs (transcript length of ≥1,000).

Although lncRNAs do not have an obvious ORF and cannot encode protein, we compared the lncRNAs and the ORF lengths of the mRNAs to discuss the differences between them. The principle of ORF analysis was based on the six-frame translation principle of nucleic acids. The distributions of the ORF lengths of the lncRNAs and mRNAs are shown in Figures 4B,C. The statistics of lncRNA and mRNA exons are shown in Figure 4D, and the statistics of lncRNA and mRNA expression levels are shown in Figure 4E. The results demonstrated that there was a significantly higher correlation between divergent lncRNAs. When the number of exons was <4, the number of exons of lncRNAs was significantly more than that of mRNAs; when the number of exons was ≥4, the number of exons of lncRNAs was significantly less than that of mRNAs. Furthermore, the identified lncRNAs tend to be shorter in ORF length than mRNAs.

### Functional Prediction of Differentially Expressed lncRNAs and Analysis of the Interaction Between lncRNAs and mRNAs

**Figure 5:**
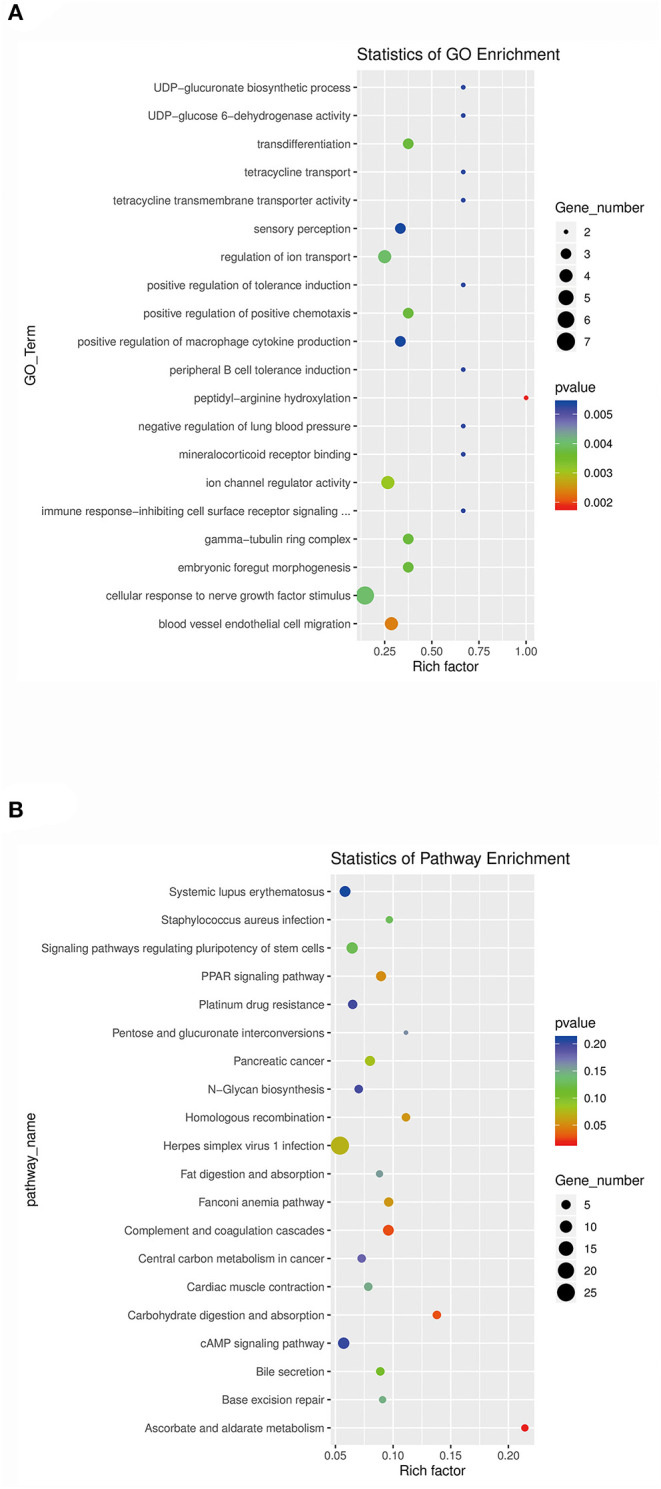
Analysis of the functional enrichment of target genes of the significantly differentially expressed lncRNAs. **(A)** GO functional enrichment analysis of the predicted target genes of the differentially expressed lncRNAs. y-axis: GO terms; x-axis: rich factor. The color of each bubble represents the *p*-value, and the bubble size represents the gene number. **(B)** KEGG pathway enrichment analysis of the predicted target genes of the differentially expressed lncRNAs. y-axis: pathway name; x-axis: rich factor. The color of each bubble represents the *p*-value, and the bubble size represents the gene number.

The target genes of lncRNAs with significantly differential expression were analyzed for functional enrichment using Gene Ontology (GO) (http://geneontology.org) and Kyoto Encyclopedia of Genes and Genomes (KEGG) (http://www.kegg.jp/kegg). GO analysis revealed that the significantly enriched GO terms of differentially expressed lncRNAs were UDP-glucuronate biosynthetic process (GO:0006065), UDP-glucose 6-dehydrogenase activity (GO:0003979), transdifferentiation (GO:0060290), tetracycline transport (GO:0015904), tetracycline transmembrane transporter activity (GO:0008493), sensory perception (GO:0007600), regulation of ion transport (GO:0043269), positive regulation of tolerance induction (GO:0002645), positive regulation of positive chemotaxis (GO:0050927), positive regulation of macrophage cytokine production (GO:0060907), peripheral B cell tolerance induction (GO:0002451), peptidyl-arginine hydroxylation (GO:0030961), negative regulation of lung blood pressure (GO:0061767), mineralocorticoid receptor binding (GO:0031962), ion channel regulator activity (GO:0099106), immune response-inhibiting cell surface receptor signaling (GO:0002767), gamma-tubulin ring complex (GO:0008274), embryonic foregut morphogenesis (GO:0048617), cellular response to nerve growth factor stimulus (GO:1990090), and blood vessel endothelial cell migration (GO:0043534); the top 20 GO enrichment terms are shown in Figure 5A. The KEGG analysis revealed that the significantly enriched pathways of differentially expressed lncRNAs were systemic lupus erythematosus (ko05322), *Staphylococcus aureus* infection (ko05150), signaling pathways regulating pluripotency of stem cells (ko04550), peroxisome proliferator-activated receptor (PPAR) signaling pathway (ko03320), platinum drug resistance (ko01524), pentose and glucuronate interconversions (ko00040), pancreatic cancer (ko05212), N-glycan biosynthesis (ko00510), homologous recombination (ko03440), herpes simplex virus 1 infection (ko05168), fat digestion and absorption (ko04975), Fanconi anemia pathway (ko03460), complement and coagulation cascades (ko04610), central carbon metabolism in cancer (ko05230), cardiac muscle contraction (ko04260), carbohydrate digestion and absorption (ko04973), cAMP signaling pathway (ko04024), bile secretion (ko04976), base excision repair (ko03410), and ascorbate and aldarate metabolism (ko00053); the top 20 KEGG enrichment pathways are shown in Figure 5B. Interestingly, swine HEV ORF3 might affect the pentose and glucuronate interconversions (ko00040) and mediate the formation of obstructive jaundice by influencing bile secretion (ko04976).

There are two kinds of regulation modes of lncRNA. One is *cis*-regulation; i.e., the lncRNA regulates the expression of its neighboring genes. According to the prediction positional relationships, the *cis*-regulatory target genes of lncRNAs were defined as a differentially expressed lncRNA and differentially expressed mRNA within 100 kb in either direction in the chromosome (31, 32). The second type of regulation is *trans*-regulation; i.e., the expression of lncRNA crosses chromosome regulatory genes and is difficult to verify in later stages. The target genes of lncRNAs with significantly differential expression were analyzed for functional enrichment using GO (http://geneontology.org) and KEGG (http://www.kegg.jp/kegg). The top 20 GO enrichment terms are shown in Figure 5A; the top five GO terms were UDP-glucuronate biosynthetic process, UDP-glucose 6-dehydrogenase activity, transdifferentiation, tetracycline transport, and tetracycline transmembrane transporter activity. The top 20 KEGG enrichment pathways are shown in Figure 5B; the top five pathways were systemic lupus erythematosus, *S. aureus* infection, signaling pathways regulating pluripotency of stem cells, the PPAR signaling pathway, and platinum drug resistance.

The significantly differentially expressed mRNAs, including 62 genes and 6,564 transcripts, were used to predict the target genes of the 124 known lncRNAs. In this study, only the upstream and downstream 100 kb mRNAs and known lncRNAs were predicted for *cis*-regulation. Finally, the target gene prediction results identified 45 lncRNAs corresponding to candidate mRNAs (Supplementary Table 1). The sequencing data of all experimental samples in the FASTQ format have been submitted to the Sequence Read Archive of NCBI under accession number GSE147129.

### qRT-PCR Validation of Partial Differentially Expressed lncRNAs

**Figure 6:**
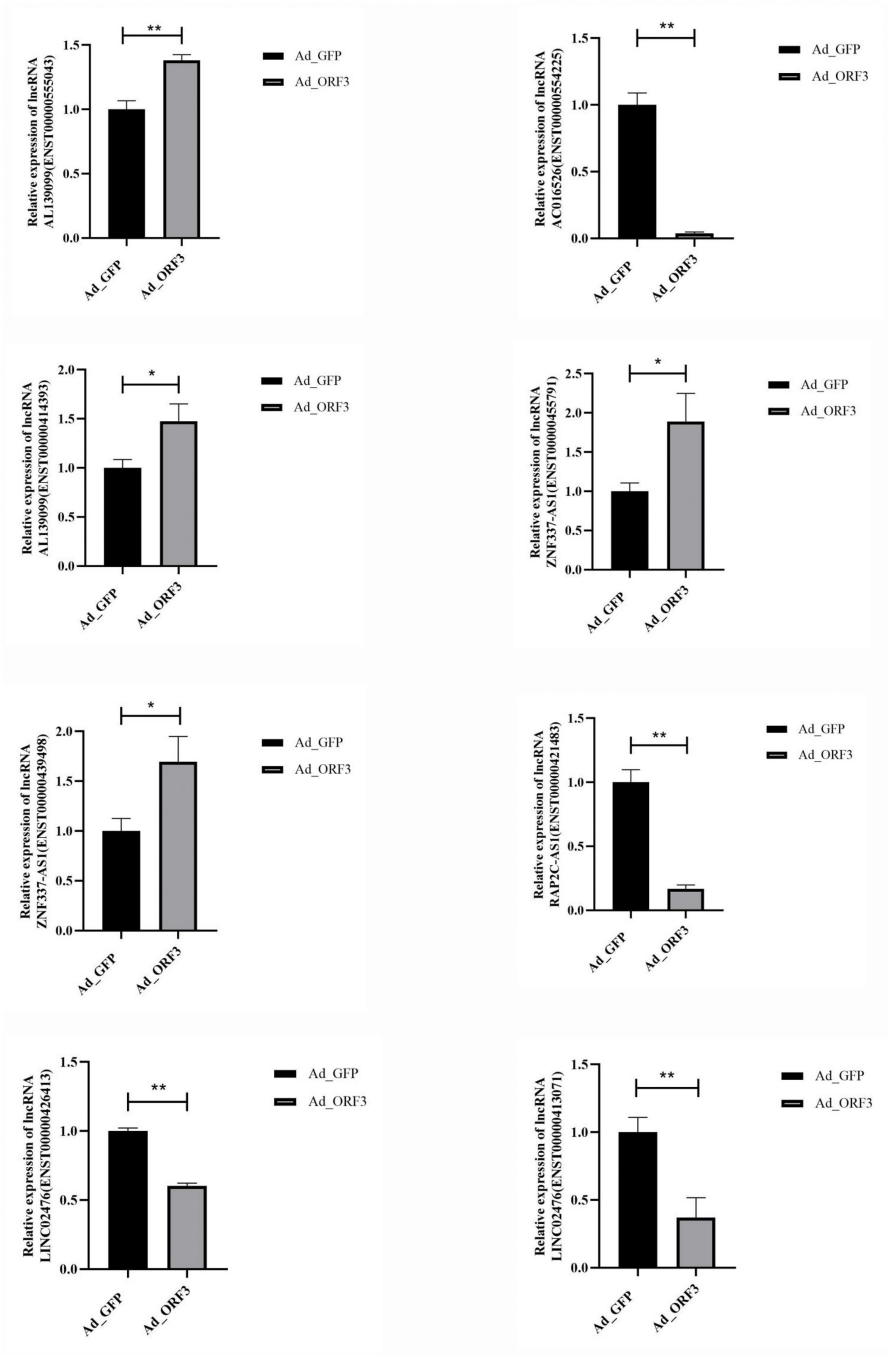
qRT-PCR validation for the eight selected significantly differentially expressed lncRNAs. *p < 0.05, **p < 0.01.

We selected 12 significantly differentially expressed lncRNAs (*p* ≤ 0.05 and *q* ≤ 1) for qRT-PCR validation, including six upregulated lncRNAs with |log2 fold-change |(|log2FC|) ≥ 2, and six downregulated lncRNAs with |log2 fold-change |(|log2FC|) ≥ 1. There were several transcripts for some lncRNAs. GAPDH was used as an internal control. The primers are listed in Table 1. These 12 lncRNAs corresponded to 21 transcripts. Among them, the upregulated lncRNAs were *PLCG1-AS1, AL139099, AC005062, FP236383, FP671120* (ENST00000627981 and ENST00000631211), and *ZNF337-AS1* (ENST00000428254, ENST00000455791, ENST00000420803, ENST00000421829, ENST00000414393, and ENST00000439498); the downregulated lncRNAs were *AP005329* (ENST00000581905 and ENST00000578800), *AP000977, LINC02476* (ENST00000431071 and ENST00000426413), *RAP2C-AS1* (ENST00000441399 and ENST00000421483), *AC107959* (ENST00000502083 and ENST00000523884), and *AC016526*. The qRT-PCR validation results showed that the expression patterns of eight lncRNAs were consistent with the sequencing data, which indicated that there were a certain number of false-positives in the high-throughput sequencing data (Figure 6 and Supplementary Table 2).

## Discussion

Swine HE is a new type of zoonotic infectious disease, which seriously threatens the development of animal husbandry and public health (33, 34). One typical clinical symptom of swine HE is jaundice, and the liver of pigs show pathological changes characteristic of hepatitis. About 1 week after infection with swine HEV, fecal detoxification begins to appear in pigs, which lasts until 55 days after infection. At 14–32 days after infection, symptoms of viremia appear. At 17–24 days after infection, anti-HEV IgM antibodies can be detected. At 3 months after infection, IgG is maintained at a high level. In infected animals, pathological sections show typical manifestations of acute hepatitis (35–37).

HEV ORF3 protein is a viral regulatory protein, which regulates many kinds of cell signaling pathways by targeting the regulatory subunits of p85α that bind to tyrosine kinase Src, phosphatidylinositol 3 kinase, phospholipase C gamma, and adaptor protein GRB2. However, ORF3 mainly activates the mitogen-activated protein kinase (MAPK) pathway through Src homology 3 domains (38). ORF3 encodes a phosphorylated protein with unclear function, which promotes the elimination of alpha (1)-microglobulin/bikunin precursor (AMBP) in hepatocytes, thus affecting their metabolism (39). ORF3 is the main factor affecting the outflow of HEV from infected cells and exists on the surface of the released HEV particles, which might be regulated by lipid metabolism (40). ORF3 is the key to infection *in vivo*, which might regulate the response of the host. The epidermal growth factor receptor (EGFR) pathway can regulate the signal transduction of endometrial growth factor, thus affecting cell survival. Signal transducer and activator of transcription 3 (STAT3) expression can also be regulated, leading to a significant reduction of the inflammatory response. These findings might provide strong support for the effect of ORF3 on virus replication (41–44). Taken together, several functions of the ORF3 protein have already been reported. ORF3 interacts with signal transduction pathway proteins by targeting proline-rich regions and Src homologous domains. Whether the EGFP-ORF3 fusion protein can still bind to these regions requires further study. lncRNAs are non-coding RNAs with a length of more than 200 nucleotides (45, 46). lncRNAs play an important role in many life activities, such as dose compensation effect, epigenetic regulation, cell cycle regulation, and cell differentiation regulation. lncRNAs have become a hot topic in genetic research (47–51).

In this study, we used transcriptome sequencing to identify the significantly differentially expressed lncRNAs and mRNAs in HepG2 cells overexpressing swine HEV ORF3. We identified 62 genes and 6,564 transcripts, including 195 novel lncRNAs and 124 known lncRNAs. Among them, we chose the 124 known lncRNAs as our study object, which would be easy to validate and functionally analyze in later research (52). lncRNA regulation can be divided into two types: *cis*-regulation (53, 54) and *trans*-regulation (31, 55). We focused on the prediction and analysis of *cis*-regulated differentially expressed mRNAs and lncRNAs.

A previous report used HepG2 and EGFP-expressing HepG2 as controls, and in response to EGFP-ORF3, transcriptome sequencing showed that *CLDN6, YLPM1, APOC3, NLRP1, SCARA3, FGA, FGG, FGB*, and *FREM1* were upregulated and that *SLC2A3, DKK1, BPIFB2*, and *PTGR1* were downregulated (56). We examined our raw data from the high-throughput sequencing and examined the 13 previously reported genes one by one. The results showed that *CLDN6, YLPM1, APOC3, NLRP1*, S*CARA3, FGB, FREM1, SLC2A3, DKK1, BPIFB2*, and *PTGR1* were present in our raw data (Supplementary Table 3); however, they did not show significantly differential expressions. FGA and FGG were not present in our raw data. The possible reasons for these discrepancies were analyzed and summarized. In our study, the latest software, StringTie, was used to assemble and quantify the reads, and edgeR was used to determine difference statistics and perform visual mapping. The highlight of our project is that we analyze the data from the gene and transcript levels. edgeR was used for differential analysis (|log2 fold change| ≥ 1 [multiple of difference > 2 times], *p* < 0.05). At the same time, we provided further definitions or suggestions for the difference threshold according to the initial operation results. Another very important reason is that the expression systems are different. Xu et al. used lentivirus-mediated overexpression of ORF3 in HepG2 cells, screened positive clones using G418, and constructed stable cell lines. However, we used adenovirus directly to mediate high-level overexpression of ORF3 in HepG2 cells. The expression level of ORF3 might affect the differentially expressed genes and their fold change in the target cells. We used adenovirus to mediate the overexpression of ORF3 in HepG2 cells. Although ORF3 is fused with EGFP, whether the function of the EGFP-ORF3 fusion protein is identical to that of native ORF3 requires further experimental verification. As reported before, transcriptome sequencing using HepG2 cells and EGFP-expressing-HepG2 cells as negative controls indicated that the expression of EGFP did not interfere with the study of ORF3 protein function. The function of lncRNAs can be predicted by the function of their target genes; therefore, the top 20 pathways were predicted by KEGG analysis and comprised systemic lupus erythematosus, *S. aureus* infection, signaling pathways regulating pluripotency of stem cells, the PPAR signaling pathway, platinum drug resistance, pentose and glucuronate interconversions, pancreatic cancer, N-glycan biosynthesis, homologous recombination, herpes simplex virus 1 infection, fat digestion and absorption, the Fanconi anemia pathway, complement and coagulation cascades, central carbon metabolism in cancer, cardiac muscle contraction, carbohydrate digestion and absorption, cAMP signaling pathway, bile secretion, base excision repair, and ascorbate and aldarate metabolism. Among them, we are very interested in the bile secretion pathway, because jaundice is the typical clinical symptom caused by swine HEV, and jaundice can only be caused if bile cannot be excreted (56–60). Bilirubin is an orange-yellow bile pigment, which is the main metabolite of iron porphyrin compounds *in vivo* and is an important basis for judging jaundice in the clinic. In the smooth ER, bilirubin and UDP-glucuronic acid are esterified under the action of glucuronosyltransferase to generate glucuronosyl bilirubin, which is then excluded from the liver together with bile under the action of Golgi bodies and lysosomes. However, bilirubin metabolism disorder leads to jaundice, and the typical clinical symptom of swine HE is jaundice (61–63). Therefore, swine HEV ORF3 might affect the pentose and glucuronate interconversions of the above top 20 pathways and mediate the occurrence of jaundice. The obstruction of the bile excretion channel leads to increased internal pressure in the bile duct and capillaries, which leads to the reverse flow of glucuronide bilirubin into the blood and the increase of serum bilirubin concentration, leading to obstructive jaundice (64–66). Therefore, swine HEV ORF3 might also mediate the formation of obstructive jaundice by influencing the bile secretion of the above top 20 pathways. These two pathways will be studied in our follow-up experiments and might represent a new direction to elucidate the pathogenesis of swine HEV.

In the present study, we identified the lncRNA and mRNA differential expression profiles in HepG2 cells overexpressing swine HEV ORF3. The structures of the lncRNAs were compared with those of the mRNAs, and the functional enrichment of target genes was analyzed using GO and KEGG, which revealed the possible functions of the lncRNAs. The qRT-PCR validation results demonstrated that the sequencing data were reliable. In the future, we will investigate the pathogenesis of swine HEV by studying the significantly differentially expressed lncRNAs and their regulatory target genes.

## Conclusion

Swine HEV ORF3 might affect the pentose and glucuronate interconversions and mediate the formation of obstructive jaundice by influencing bile secretion. Our findings are of great significance in revealing the function of swine HEV ORF3 and explaining the molecular mechanism of swine HEV pathogenesis.

## Data Availability Statement

The datasets presented in this study can be found in online repositories. The names of the repository/repositories and accession number(s) can be found at: NCBI SRA; GSE147129.

## Author Contributions

HJ, QH, XS, ZS, and YL contributed to the conception and design of the study. HJ, ZZ, and YZ contributed to data acquisition and data analysis. BL, GG, and ML contributed to data interpretation. HJ, HZ, YX, and XG drafted the manuscript. HJ, WL, and LG revised the manuscript. All authors have read and approved the final version of the manuscript.

## Conflict of Interest

The authors declare that the research was conducted in the absence of any commercial or financial relationships that could be construed as a potential conflict of interest.
