# Supplementary figures and images for: Deep Insight Into Long Non-coding RNA and mRNA Transcriptome Profiling in HepG2 Cells Expressing Genotype IV Swine Hepatitis E Virus ORF3
Source: Front Vet Sci. 2021 Apr 29;8:625609. doi: 10.3389/fvets.2021.625609 (PMC8116512; doi:10.3389/fvets.2021.625609)

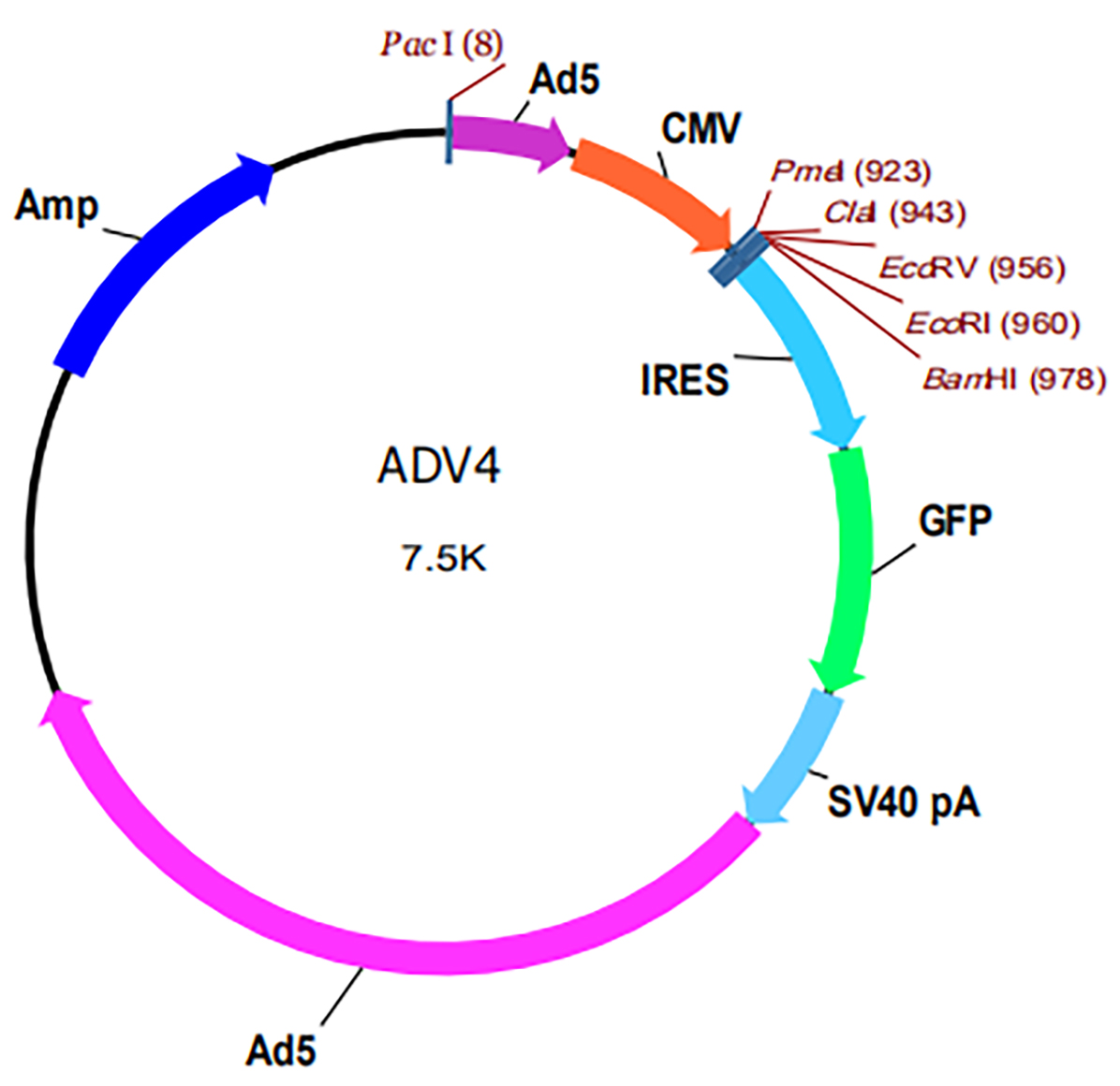

Supplement: Supplementary file 1 [file Image_1.JPEG]

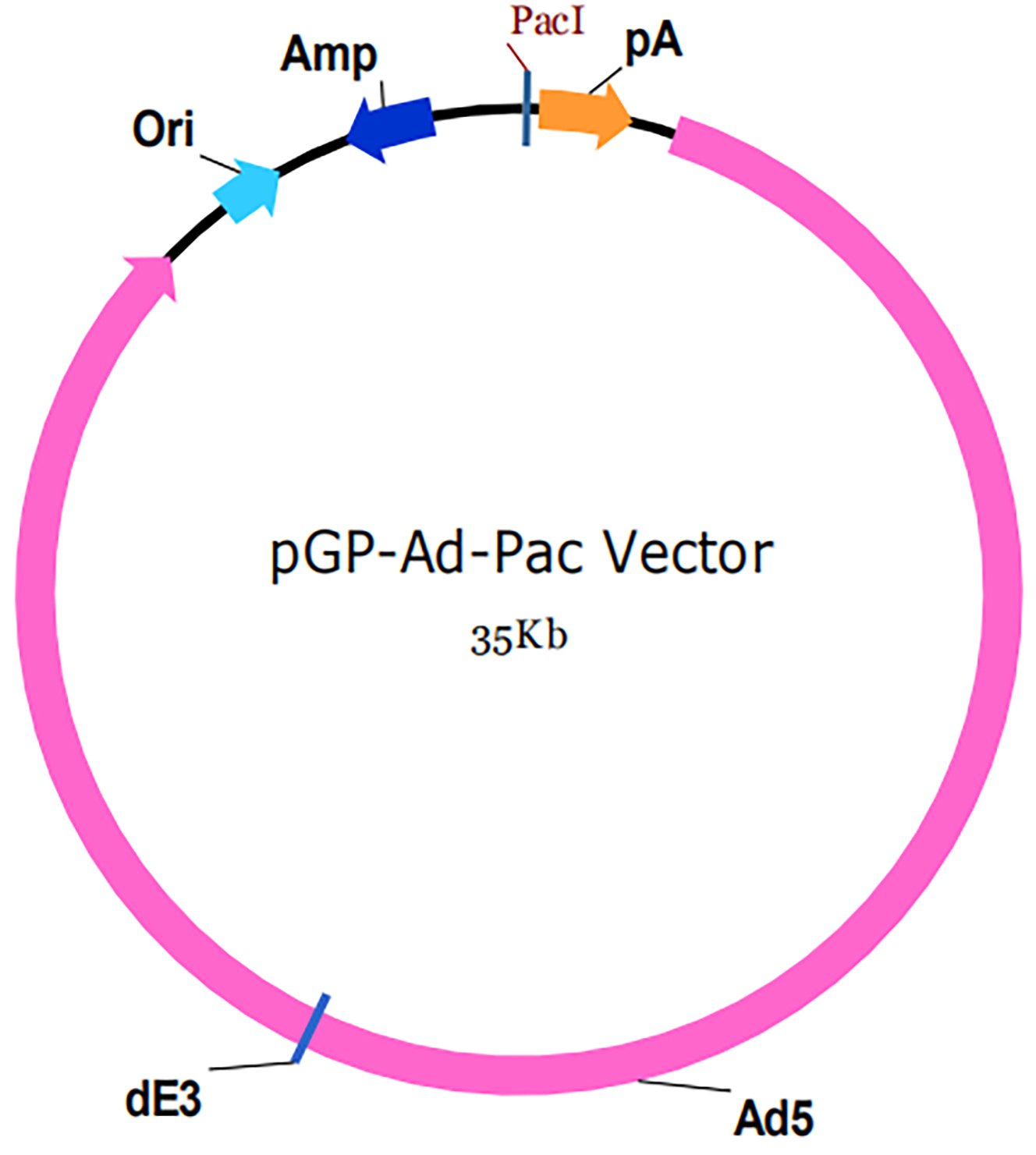

Supplement: Supplementary file 2 [file Image_2.JPEG]
