# Supplementary material for: Deep Insight Into Long Non-coding RNA and mRNA Transcriptome Profiling in HepG2 Cells Expressing Genotype IV Swine Hepatitis E Virus ORF3
Source: Front Vet Sci. 2021 Apr 29;8:625609. doi: 10.3389/fvets.2021.625609 (PMC8116512; doi:10.3389/fvets.2021.625609)
Supplement: Supplementary file 3 [file Data_Sheet_1.docx]

**SUPPLEMENTARY TABLE 1** **|** The 45 known lncRNAs corresponding to candidate mRNAs predicted by *cis*-regulation.

|  |  | Group1  Ad_ORF3 | Group2  Ad_GFP |  |  |  |
| --- | --- | --- | --- | --- | --- | --- |
| lncRNA_Gene Name | transcript_name | FPKM | FPKM | Log2 (G1/G2) | Target genes name | Target transcript name |
| AL137002 | ENST00000639766 | 0.735705333 | 0 | Inf | F10 | ENST00000409306 |
|  |  |  |  |  | MCF2L | ENST00000397017 |
|  |  |  |  |  | CUL4A | ENST00000375440 |
|  |  |  |  |  | MCF2L | ENST00000261963 |
|  |  |  |  |  | CUL4A | ENST00000375441 |
| KCTD13 | ENST00000567795 | 1.037026667 | 0.014848667 | 6.13 | KCTD13 | ENST00000649581 |
|  |  |  |  |  | TAOK2 | ENST00000543033 |
|  |  |  |  |  | INO80E | ENST00000567705 |
|  |  |  |  |  | KCTD13 | ENST00000561540 |
| AL049840 | ENST00000498989 | 1.214969333 | 0.005609 | 7.76 | KLC1 | ENST00000246489 |
|  |  |  |  |  | KLC1 | ENST00000554280 |
|  |  |  |  |  | XRCC3 | ENST00000554811 |
|  |  |  |  |  | KLC1 | ENST00000452929 |
| AL513327 | ENST00000587696 | 0.474744333 | 0 | inf | PHC2 | ENST00000485928 |
| NR2F1-AS1 | ENST00000606696 | 0 | 0.221982333 | -inf | NR2F1 | ENST00000644230 |
| GAS5 | ENST00000432536 | 3.37027 | 0.554627333 | 2.60 | DARS2 | ENST00000649067 |
|  |  |  |  |  |  | ENST00000648807 |
|  |  |  |  |  |  | ENST00000471476 |
|  |  |  |  |  |  | ENST00000647645 |
| ABALON | ENST00000629058 | 0.509683667 | 5.709641667 | -3.49 | BCL2L1 | ENST00000456404 |
| AC105760 | ENST00000418430 | 0.078220333 | 0.527274667 | -2.75 | COPS8 | ENST00000392008 |
| SNHG20 | ENST00000434411 | 0.122156333 | 0.684904667 | -2.49 | SNHG20 | ENST00000647734 |
|  |  |  |  |  | SEC14L1 | ENST00000589827 |
| AC092683 | ENST00000610408 | 0.383539 | 0.050409667 | 2.93 | ANKRD36B | ENST00000359901 |
| BAIAP2-DT | ENST00000577066 | 0.241292333 | 0.863663333 | -1.84 | BAIAP2 | ENST00000575712 |
|  |  |  |  |  |  | ENST00000428708 |
|  |  |  |  |  |  | ENST00000575245 |
| UGDH-AS1 | ENST00000504032 | 0.175701667 | 0.715943 | -2.03 | UGDH | ENST00000514106 |
| AL139099 | ENST00000555043 | 0.729860333 | 0.135690333 | 2.43 | RPL36AL | ENST00000298289 |
| PAXBP1-AS1 | ENST00000458479 | 0.226293667 | 0.040570667 | 2.48 | PAXBP1 | ENST00000466846  ENST00000331923 |
| NOP14-AS1 | ENST00000512712 | 0.371596667 | 0.095471667 | 1.96 | MFSD10 | ENST00000507272 |
| AC020907 | ENST00000586871 | 6.264388667 | 0.972675667 | 2.69 | FXYD1 | ENST00000455515 |
|  |  |  |  |  |  | ENST00000588607 |
| AC138035 | ENST00000604669 | 1.203726333 | 0.367102667 | 1.71 | RACK1 | ENST00000626067 |
|  |  |  |  |  | RACK1 | ENST00000511473 |
|  |  |  |  |  | TRIM52 | ENST00000611618 |
|  |  |  |  |  | RACK1 | ENST00000515417 |
|  |  |  |  |  | TRIM52 | ENST00000513146 |
|  |  |  |  |  | RACK1 | ENST00000512968 |
|  |  |  |  |  | TRIM41 | ENST00000315073 |
|  |  |  |  |  | RACK1 | ENST00000508044 |
| SNHG29 | ENST00000481027 | 8.227163333 | 21.882879 | -1.41 | TRPV2 | ENST00000338560 |
| PSMA3-AS1 | ENST00000556002 | 0.287958 | 1.061348667 | -1.88 | ARID4A | ENST00000395168 |
|  |  |  |  |  | ARID4A | ENST00000431317 |
|  |  |  |  |  | PSMA3 | ENST00000412908 |
|  |  |  |  |  | PSMA3 | ENST00000557087 |
| SLX1B-SULT1A4 | ENST00000344620 | 0.928187333 | 2.544404 | -1.45 | SMG1P6 | ENST00000532337 |
|  |  |  |  |  | SULT1A4 | ENST00000360423 |
| GAS5 | ENST00000455838 | 0.925727 | 3.166005667 | -1.77 | DARS2 | ENST00000649067 |
|  |  |  |  |  |  | ENST00000648807 |
|  |  |  |  |  |  | ENST00000471476 |
|  |  |  |  |  |  | ENST00000647645 |
| SNHG29 | ENST00000480811 | 1.378864 | 0.595982333 | 1.21 | TRPV2 | ENST00000338560 |
| RAD51-AS1 | ENST00000499988 | 2.132966667 | 0.732431667 | 1.54 | RAD51 | ENST00000267868 |
|  |  |  |  |  |  | ENST00000525066 |
| AL139011 | ENST00000642063 | 0.470216667 | 1.728875333 | -1.88 | COPA | ENST00000648805 |
|  |  |  |  |  | NCSTN | ENST00000424645 |
|  |  |  |  |  | COPA | ENST00000647693 |
|  |  |  |  |  | COPA | ENST00000368069 |
|  |  |  |  |  | COPA | ENST00000649963 |
|  |  |  |  |  | COPA | ENST00000649676 |
| MAPKAPK5-AS1 | ENST00000428207 | 0.564660667 | 1.280113 | -1.18 | TMEM116 | ENST00000550831 |
|  |  |  |  |  | TMEM116 | ENST00000355445 |
|  |  |  |  |  | ALDH2 | ENST00000548536 |
|  |  |  |  |  | TMEM116 | ENST00000549537 |
| NOP14-AS1 | ENST00000515194 | 0.263367 | 0.626247667 | -1.25 | MFSD10 | ENST00000507272 |
| SNHG29 | ENST00000487066 | 1.031835 | 0.395523 | 1.38 | TRPV2 | ENST00000338560 |
| FIRRE | ENST00000427391 | 0.542693333 | 0.267472 | 1.02 | FIRRE | ENST00000649541 |
| ZFAS1 | ENST00000450535 | 1.924290667 | 0.947419333 | 1.02 | ZNFX1 | ENST00000371752 |
| AL118506 | ENST00000601296 | 9.256478 | 3.653301667 | 1.34 | DNAJC5  DNAJC5 | ENST00000470551 |
|  |  |  |  |  |  | ENST00000360864 |
|  |  |  |  |  | TPD52L2  TPD52L2 | ENST00000615907 |
|  |  |  |  |  |  | ENST00000611972 |
| SNHG29 | ENST00000481898 | 0.738420333 | 2.089146667 | -1.50 | TRPV2 | ENST00000338560 |
| TPT1-AS1 | ENST00000519454 | 1.114670667 | 0.512992667 | 1.12 | TPT1 | ENST00000616577 |
| HCP5 | ENST00000414046 | 0.357644333 | 1.116246333 | -1.64 | MICB | ENST00000252229 |
| ARMCX5-GPRASP2 | ENST00000476910 | 0.162922667 | 0.539905 | -1.73 | ARMCX5 | ENST00000372742 |
|  |  |  |  |  | GPRASP1 | ENST00000652542 |
| NOP14-AS1 | ENST00000505731 | 0.097266333 | 0.279495667 | -1.52 | MFSD10 | ENST00000507272 |
| AC068888 | ENST00000546793 | 0.211536 | 0.531066 | -1.33 | EIF4B | ENST00000552490 |
|  |  |  |  |  |  | ENST00000550704 |
|  |  |  |  |  | TNS2 | ENST00000549311 |
|  |  |  |  |  | EIF4B | ENST00000551527 |
| NUP50-DT | ENST00000609284 | 1.380662 | 0.642966667 | 1.10 | NUP50 | ENST00000347635 |
| EIF3J-DT | ENST00000560049 | 1.069893667 | 0.479940667 | 1.16 | SPG11 | ENST00000558319 |
| AL513327 | ENST00000630229 | 0.513033667 | 1.471302333 | -1.52 | PHC2 | ENST00000485928 |
| AL928654 | ENST00000504332 | 0.749179667 | 0.309918667 | 1.27 | TEDC1 | ENST00000392522 |
|  |  |  |  |  | PACS2 | ENST00000547903 |
|  |  |  |  |  | TEDC1 | ENST00000392523 |
|  |  |  |  |  | CRIP1 | ENST00000496700 |
|  |  |  |  |  | TEDC1 | ENST00000354560 |
|  |  |  |  |  | CRIP2 | ENST00000538259 |
| NR2F1-AS1 | ENST00000504474 | 0.251937 | 0.105784667 | 1.25 | NR2F1 | ENST00000644230 |
| AC020978 | ENST00000571197 | 5.545824 | 11.64993967 | -1.07 | SLC7A6 | ENST00000563080 |
|  |  |  |  |  | SLC7A6 | ENST00000648130 |
|  |  |  |  |  | ESRP2 | ENST00000566774 |
| SP2-AS1 | ENST00000451140 | 0.215777 | 0.661411667 | -1.62 | MRPL10 | ENST00000414011 |
|  |  |  |  |  | LRRC46 | ENST00000269025 |
|  |  |  |  |  | MRPL10 | ENST00000290208 |
|  |  |  |  |  | SP2 | ENST00000376741 |
| STAG3L5P-PVRIG2P-PILRB | ENST00000310771 | 0.0833 | 0.180638333 | -1.12 | MEPCE | ENST00000414441 |
|  |  |  |  |  | PILRB | ENST00000448382 |
|  |  |  |  |  | PPP1R35 | ENST00000487452 |
|  |  |  |  |  | PVRIG2P | ENST00000435460 |
| UBL7-AS1 | ENST00000499217 | 0.308199333 | 0.673085333 | -1.13 | UBL7 | ENST00000565130 |
|  |  |  |  |  |  | ENST00000564488 |

**SUPPLEMENTARY TABLE 2** **|** The information for the eight lncRNAs validated using qRT-PCR.

| lncRNA_Gene Name | transcript_name | Fold-change  Ad_ORF3(FPKM)/Ad_GFP(FPKM) | Log2 (fold -change) |
| --- | --- | --- | --- |
| LINC02476 | ENST00000431071 | 0.43 | -1.21 |
| LINC02476 | ENST00000426413 | 0.43 | -1.21 |
| RAP2C-AS1 | ENST00000441399 | 0.46 | -1.11 |
| AC016526 | ENST00000554225 | 0.49 | -1.04 |
| AL139099 | ENST00000555043 | 5.38 | 2.43 |
| ZNF337-AS1 | ENST00000455791 | 2.76 | 1.46 |
| ZNF337-AS1 | ENST00000414393 | 2.76 | 1.46 |
| ZNF337-AS1 | ENST00000439498 | 2.76 | 1.46 |

**SUPPLEMENTARY TABLE 3** **|** The information of the 13 previously reported genes from our the high-throughput sequencing raw data.

| Gene Short Name | p-value | Ad_ORF3  FPKM | Ad_GFP  FPKM | Log2 fold change | significant |
| --- | --- | --- | --- | --- | --- |
| FGG | 0.79 | 87.68 | 87.15 | 0.01 | no |
| FGA | 1.03 | 130.39 | 126.81 | 0.04 | no |
| APOC3 | 0.95 | 86.53 | 91.24 | -0.08 | no |
| SLC2A3 | 0.98 | 78.34 | 79.62 | -0.02 | no |
| DKK1 | 0.87 | 34.45 | 39.51 | -0.20 | no |
| KRT19 | 0.82 | 0.18 | 0.22 | -0.29 | no |
| BPIFB2 | 1.01 | 252.64 | 250.42 | 0.01 | no |
| CLDN6 | 0.82 | 2.48 | 3.02 | -0.29 | no |
| FREM1 | 0.96 | 5.70 | 5.95 | -0.06 | no |
| YLPM1 | 1.00 | 6.41 | 6.43 | 0.00 | no |
| SCARA3 | 1.03 | 2.15 | 2.08 | 0.05 | no |
| NLRP1 | 0.82 | 0.18 | 0.22 | -0.29 | no |
| PTGR1 | 0.97 | 3.16 | 3.26 | -0.05 | no |

^*^ FPKM: Fragment Per Kilobase of Exon Per Million Mapped Fragments.
